# Inferring interactions in assemblies of stochastic integrate-and-fire neurons from spike recordings: method, applications and software

**DOI:** 10.1186/1471-2202-12-S1-P40

**Published:** 2011-07-18

**Authors:** Carlo Barbieri, Simona Cocco, Rémi Monasson

**Affiliations:** 1 Simons Center for Systems Biology, Institute for Advanced Study, Princeton, New Jersey, USA; 2 CNRS-Laboratoire de Physique Statistique de l'Ecole Normale Superieure, Paris 5e, France; 3 CNRS-Laboratoire de Physique Theorique de l'Ecole Normale Superieure, Paris 5e, France

## 

Multi-electrode recordings make possible the simultaneous spiking activity of tens of neurons for hours. An important issue is to reconstruct the network of functional interactions between the cells from the observed correlated activity. We have recently designed a fast inference method for this purpose, in which the cells are modeled as Leaky Integrate-and-Fire (LIF) neurons, coupled through a set of interactions Jij [1,2]. Each LIF neuron i receives a stochastic current, equal to the sum of an external current I_i_ and of a Gaussian white noise process with variance *s2*.

The LIF model implicitly defines the likelihood P of the spiking times {tj,k} given the currents Ii and synaptic interactions Jij. Given the spiking times {tj,k} we infer the couplings and currents by maximizing P (a priori information can be considered, too, see below). Though P can be, in principle, calculated through the numerical resolution of the Fokker-Planck equation associated to the LIF dynamical equations [3], this approach is too slow to treat data sets with tens of neurons and hundreds of thousands of spikes in a reasonable time. In our approach we approximate P from the contribution coming from the most probable trajectory for the potential for each cell i, referred to as Vi*(t). This approximation is exact when the amplitude *s* of the noise is small. The determination of Vi*(t) was done numerically by Paninski for one cell in [4]. We have found an exact procedure to determine Vi*(t) analytically in a time growing linearly with the number of spikes and quadratically with the number of neurons, which allows us to process very large recordings [2]. Once the most probable trajectory for the potential has been determined for a set of interactions and currents, we calculate the corresponding log-likelihood and maximize it over the interactions and the currents using convex optimizations methods.

Our algorithm has been tested again artificially generated data (with up to 160 cells, 20 millions spikes), and real experimental data (recordings of 32 to 60 ganglion cells in the salamander retina, data courtesy of M. Meister) [1,2]. As an example, it takes us about 30 seconds to process a set of 120,000 spikes fired by 32 cells on a commercial desktop computer.

We present a software package, running in C, Matlab and Mathematica, offering a practical implementation of our inference method. The program requires as an input the list of spiking times and cell numbers. The user can set various parameters of the model or choose to infer them from the data. A choice of priors over the interactions (based on the L_1_ and L_2_ norms) are proposed. Various optimization routines, making use or not of the second derivatives of the log-likelihood are available. The output of the program is the matrix of interactions J_ij_, with the currents I_i_ on the diagonal, and the error bars (statistical uncertainties) on those most likely values.
